# Role of cullin-elonginB-elonginC E3 complex in bovine immunodeficiency virus and maedi-visna virus Vif-mediated degradation of host A3Z2-Z3 proteins

**DOI:** 10.1186/s12977-014-0077-9

**Published:** 2014-09-12

**Authors:** Jingyao Zhang, Jiaxin Wu, Weiran Wang, Hui Wu, Bin Yu, Jiawen Wang, Mingyu Lv, Xiaodan Wang, Haihong Zhang, Wei Kong, Xianghui Yu

**Affiliations:** National Engineering Laboratory for AIDS Vaccine, Changchun, Jilin Province People’s Republic of China; Key Laboratory for Molecular Enzymology and Engineering, the Ministry of Education, School of Life Sciences, Jilin University, No. 2699 Qianjin Street, Changchun, Jilin Province People’s Republic of China

**Keywords:** E3 ubiquitin ligase, BIV Vif, MVV Vif, CCHC motif

## Abstract

**Background:**

All lentiviruses except equine infectious anemia virus (EIVA) antagonize antiviral family APOBEC3 (A3) proteins of the host through viral Vif proteins. The mechanism by which Vif of human, simian or feline immunodeficiency viruses (HIV/SIV/FIV) suppresses the corresponding host A3s has been studied extensively.

**Results:**

Here, we determined that bovine immunodeficiency virus (BIV) and maedi-visna virus (MVV) Vif proteins utilize the Cullin (Cul)-ElonginB (EloB)-ElonginC (EloC) complex (BIV Vif recruits Cul2, while MVV Vif recruits Cul5) to degrade *Bos taurus* (bt)A3Z2-Z3 and *Ovis aries* (oa)A3Z2-Z3, respectively, via a proteasome-dependent but a CBF-β-independent pathway. Mutation of the BC box in BIV and MVV Vif, C-terminal hydrophilic replacement of btEloC and oaEloC and dominant-negative mutants of btCul2 and oaCul5 could disrupt the activity of BIV and MVV Vif, respectively. While the membrane-permeable zinc chelator TPEN could block BIV Vif-mediated degradation of btA3Z2-Z3, it had minimal effects on oaA3Z2-Z3 degradation induced by MVV Vif, indicating that Zn is important for the activity of BIV Vif but not MVV Vif. Furthermore, we identified a previously unreported zinc binding loop [C-x_1_-C-x_1_-H-x_19_-C] in the BIV Vif upstream BC box which is critical for its degradation activity.

**Conclusions:**

A novel zinc binding loop was identified in the BIV Vif protein that is important for the E3 ubiquination activity, suggesting that the degradation of btA3Z2-Z3 by BIV and that of oaA3Z2-Z3 by MVV Vif may need host factors other than CBF-β.

## Background

Lentiviruses, a subfamily of retroviruses, cause slow infections in humans and animals. Human immunodeficiency virus type 1 (HIV-1), simian immunodeficiency virus (SIV), caprine arthritis-encephalitis virus (CAEV), feline immunodeficiency virus (FIV), bovine immunodeficiency virus (BIV), maedi-visna virus (MVV) and equine infectious anemia virus (EIAV) are lentiviruses that infect humans, monkeys, goats, cats, cattle, sheep and horses, respectively. Except for EIAV, all lentiviruses require the accessory protein viral infectivity factor (Vif) to establish persistent infection and pathogenesis *in vivo* [1]. The Vif protein counteracts the antiviral activities of the apolipoprotein B mRNA-editing enzyme, catalytic polypeptide-like 3 (APOBEC3 or A3) proteins of the host [2]. These A3 proteins possess broad antiviral activities for many viruses as natural host restriction factors [3-7]. Among the A3 proteins, A3G is the most intensively studied. In the late stage of viral infection, A3G proteins are packaged into virions and induce dC to dU mutations in newly synthesized minus-strand viral DNA. These mutations cause abnormal expression of viral proteins, resulting in disruptions of the viral life cycle [8-10]. The HIV-1 accessory factor Vif plays a critical role in maintaining efficient viral replication in non-permissive cell lines [11]. HIV-1 Vif antagonizes the antiviral activity of the cellular protein A3G by recruiting the transcription cofactor CBF-β and ElonginB (EloB)-ElonginC (EloC) to the Cullin5 (Cul5)-Rbx complex to degrade A3G [3,12-18]. The functional domains that Vif uses to form the E3 ligase complex have been reported. The main sites involved in the interaction with A3G and CBF-β are in the N-terminal region of Vif [19-23]. The H-x_5_-C-x_17– 18_-C-x_3 –5_-H motif (i.e., HCCH zinc finger) and the PPLPx4L motif (also known as the Cul5 box) in the C-terminal region of HIV-1 Vif mediate selective binding to Cul5 [24-26]. Meanwhile, another C-terminal SLQ(Y/F) LA motif (BC box) downstream of the HCCH domain binds with EloC to assemble the E3 ligase complex [12,27,28]. Mechanisms of the degradation of APOBEC3 proteins induced by SIV Vif and FIV Vif also have been well studied. SIVmac239 Vif recruits the transcription cofactor CBF-β and EloB-EloC to the Cul5-Rbx complex, forming the CBF-β-Cul5-EloB-EloC E3 ubiquitin ligase to degrade the cellular antiviral protein A3G [29,30]. FIV Vif interacts with feline Cul5, EloB and EloC to form an E3 complex to induce degradation of fA3s [31].

BIV affects the immune system like many other lentiviruses [32,33], and its name was based on similarities to HIV-1 in genetic, structural, antigenic and biological factors. BIV infects cattle and causes significant but non-persistent infiltrating lymphocytes and follicular hyperplasia in the hemolymph nodes [34]. MVV is also a lentivirus which causes slowly progressive meningoencephalomyelitis and pneumonia in sheep [35]. The Vif proteins of BIV and MVV are both indispensable for viral infectivity [36]. The artiodactyl A3 proteins have been reported to have an active N-terminal DNA cytosine deaminase domain, which displays a dinucleotide deamination preference [37]. According to the nonprimate A3 nomenclature, there are four *Bos taurus* A3 (btA3) proteins: btA3Z1, btA3Z2, btA3Z3, btA3Z2-Z3 and four *Ovis aries* A3 (oaA3) proteins: oaA3Z1, oaA3Z2, oaA3Z3 and oaA3Z2-Z3. Among the A3 proteins, A3Z2-Z3 is the only double domain protein that displays fully intact levels of lentivirus restriction and is neutralized by Vif from several different species [38]. BIV and MVV Vif are known to degrade the host A3 proteins to antagonize their antiviral activity. However, whether the mechanism by which Vif of BIV and MVV neutralize the btA3s and oaA3s, respectively, is similar to that of HIV-1 Vif against human A3G remains an open question.

In our study, we chose btA3Z2-Z3 and oaA3Z2-Z3 as the target proteins to investigate the mechanism of their degradation by BIV Vif and MVV Vif proteins, respectively. Interactions of BIV and MVV Vif with Cul, EloB, EloC and/or CBF-β as part of an E3 ubiquitin ligase complex in the corresponding host cells also were examined. BIV Vif was shown to specifically interact with btCul2 (and MVV Vif with oaCul5). The function of BIV Vif and its interaction with Cul2 were explored further by mutations of the C-x_1_-C-x_1_-H-x_19_-C (CCHC) motif which may be a novel zinc finger (18, 46). Homology modeling results showed that this CCHC motif is likely a zinc binding loop. Together, results of this study indicate that BIV and MVV Vifs bind with SOCS proteins in a novel manner to form Elo-Cul-SOCS box (ECS) complexes, which may facilitate future studies of virus-host interactions.

## Results

### BIV and MVV Vifs degrade btA3Z2-Z3 and oaA3Z2-Z3, respectively, via a proteasomal pathway and affect the synthesis rate rather than the stability of these A3 proteins

**Figure 1:**
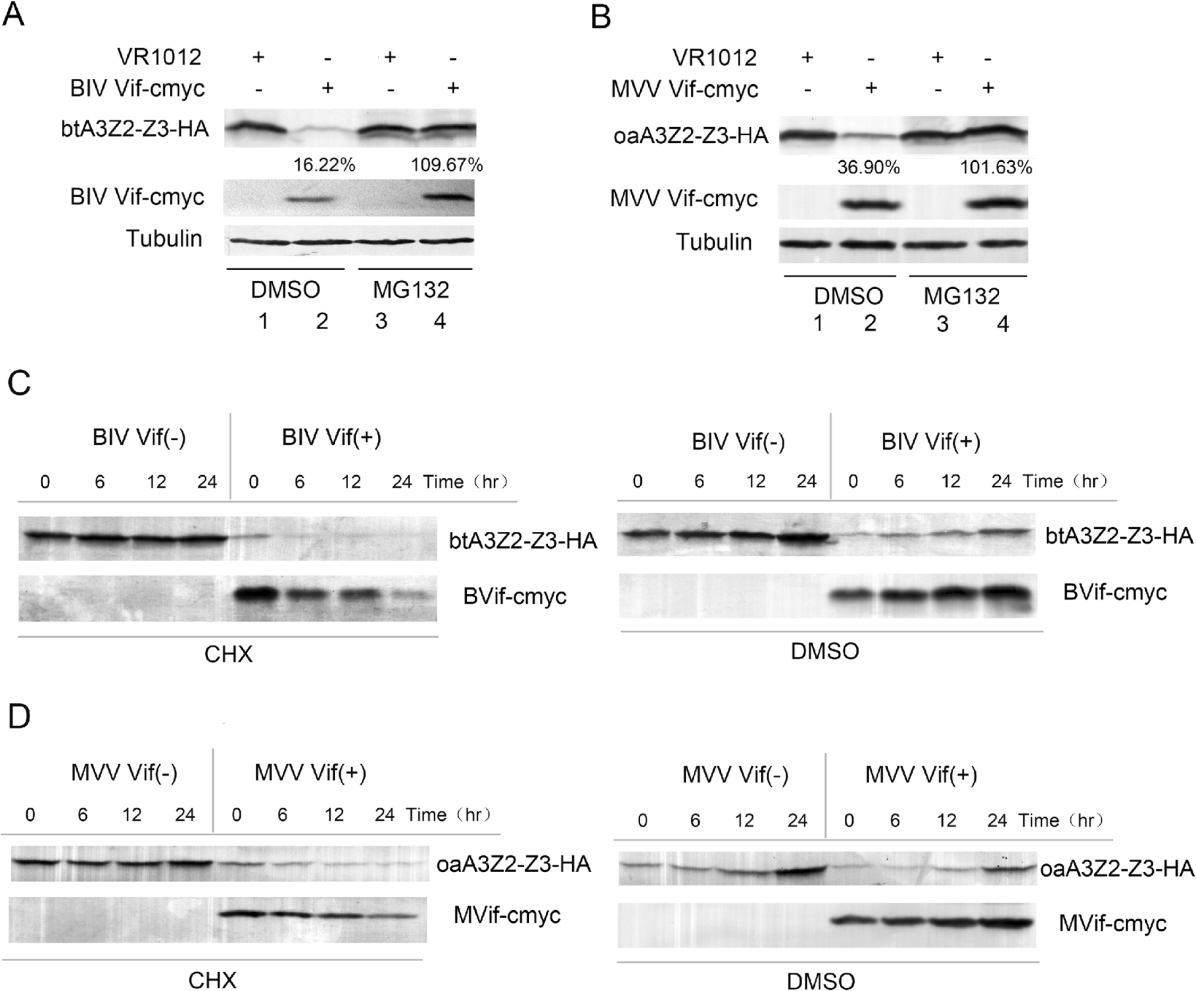
**BIV/MVV Vif affect the synthesis rate of btA3Z2-Z3/oaA3Z2-Z3, respectively, via a proteasomal pathway. (A, B)** 293 T cells (0.5 × 10^6^) were transfected with HA-tagged btA3Z2-Z3 (30 ng) or oaA3Z2-Z3 (15 ng) and cmyc-tagged BIV Vif (200 ng) or MVV Vif (200 ng) or VR1012. After 36 h of transfection, the cells were treated with the proteasome inhibitor MG132 (10 μM) (lanes 3, 4) or control DMSO (lanes 1, 2). At 48 h after transfection, the cells were harvested for Western blotting using anti-HA, anti-cmyc and anti-tubulin antibodies. Percentages of degradation with DMSO or MG132 treatment were calculated. **(C, D)** 293 T cells (0.5 × 10^6^) were transfected with HA-tagged btA3Z2-Z3 (30 ng) or oaA3Z2-Z3 (15 ng) and cmyc-tagged BIV Vif (200 ng) or MVV Vif (200 ng) or VR1012. After 18 h of transfection, the cells were treated with the protein synthesis inhibitor CHX (100 μg/ml) or DMSO as control and then harvested at the indicated time points for Western blot analysis using anti-HA and anti-cmyc antibodies. Percentages of A3 in the presence of Vif relative to that in the absence of Vif with DMSO or MG132 treatment were calculated. All degradation experiments were repeated five times. The mean value ± SEM of the remaining btA3Z2-Z3 precentage after degradation is 18.5 ± 5.2% (*P* < 0.01); The mean value and SEM of the remaining oaA3Z2-Z3 precentage after degradation is 27.8% ± 6.4% (*P* < 0.01).

Human A3G is known to be degraded by HIV-1 Vif in a proteasome-dependent manner. Studies in recent years have shown that BIV and MVV Vifs can degrade btA3Z2-Z3 and oaA3Z2-Z3, respectively, which allows for effective infection by the corresponding virus (BIV or MVV). However, the molecular mechanism by which BIV Vif degrades btA3Z2-Z3 or MVV Vif degrades oaA3Z2-Z3 is still unclear. To determine whether the degradation of btA3Z2-Z3 by BIV Vif is proteasome-dependent, 293 T cells were co-transfected with HA-tagged btA3Z2-Z3 and cmyc-tagged BIV Vif or VR1012 and treated with the proteasome inhibitor MG132 [39] or DMSO as a negative control. The results showed that btA3Z2-Z3 could be degraded by BIV Vif (Figure 1A, lane 2). However, MG132 blocked the degradation of btA3Z2-Z3 and stabilized the BIV Vif protein (Figure 1A, lane 4), implying that the degradation of btA3Z2-Z3 protein depends on proteasome activity similar to HIV-1 Vif. When 293 T cells were co-transfected with HA-tagged oaA3Z2-Z3 and cmyc-tagged MVV Vif or VR1012 and treated with the proteasome inhibitor MG132 or DMSO as a negative control, the experimental results were nearly the same as those above (Figure 1B). These findings imply that BIV Vif degrades btA3Z2-Z3 and MVV Vif degrades oaA3Z2-Z3 via a proteasomal pathway, and inhibiting this process would increase the expression of these APOBEC3 proteins to varying degrees.

To assess the steady-state level of cell-associated A3Z2-Z3 in the absence or presence of Vif, the cycloheximide (CHX, protein synthesis inhibitor) stability assay was performed (Figure 1C, D). Two sets of experiments were carried out. 293 T cells were co-transfected with HA-tagged btA3Z2-Z3 (30 ng) or HA-tagged oaA3Z2-Z3 (15 ng) and cmyc-tagged BIV Vif, cmyc-tagged MVV Vif (200 ng) or VR1012. After 18 h of transfection, transfected cells in the first set were treated with CHX, while those in the second set were treated with DMSO as a control. We then checked the expression level of A3Z2-Z3 in the absence or presence of Vif in these two sets of cells at various timepoints up to 24 h after CHX or DMSO treatment. With the addition of CHX, the synthesis of Vif and A3Z2-Z3 was inhibited, and most of the pre-existing A3Z2-Z3 was degraded by Vif, Meanwhile, Vif was partially degraded by the EloB-EloC-Cul E3 ligase. In the DMSO-treated set of cells in which protein synthesis was not inhibited, quantities of A3Z2-Z3 and Vif increased over time (compare A3Z2-Z3 levels at 0 h, 6 h, 12 h and 24 h in the presence of Vif in Figure 1C, D). Under this condition, A3Z2-Z3 was still remarkably degraded by Vif (compare A3Z2-Z3 levels in the absence and presence of Vif in Figure 1C, D). These results revealed that BIV and MVV Vifs could efficiently degrade btA3Z2-Z3 and oaA3Z2-Z3, respectively.

### BIV Vif combines with Cul2, EloB and EloC and MVV Vif combines with Cul5, EloB and EloC to induce proteasomal degradation of btA3Z2-Z3 and oaA3Z2-Z3, respectively

**Figure 2:**
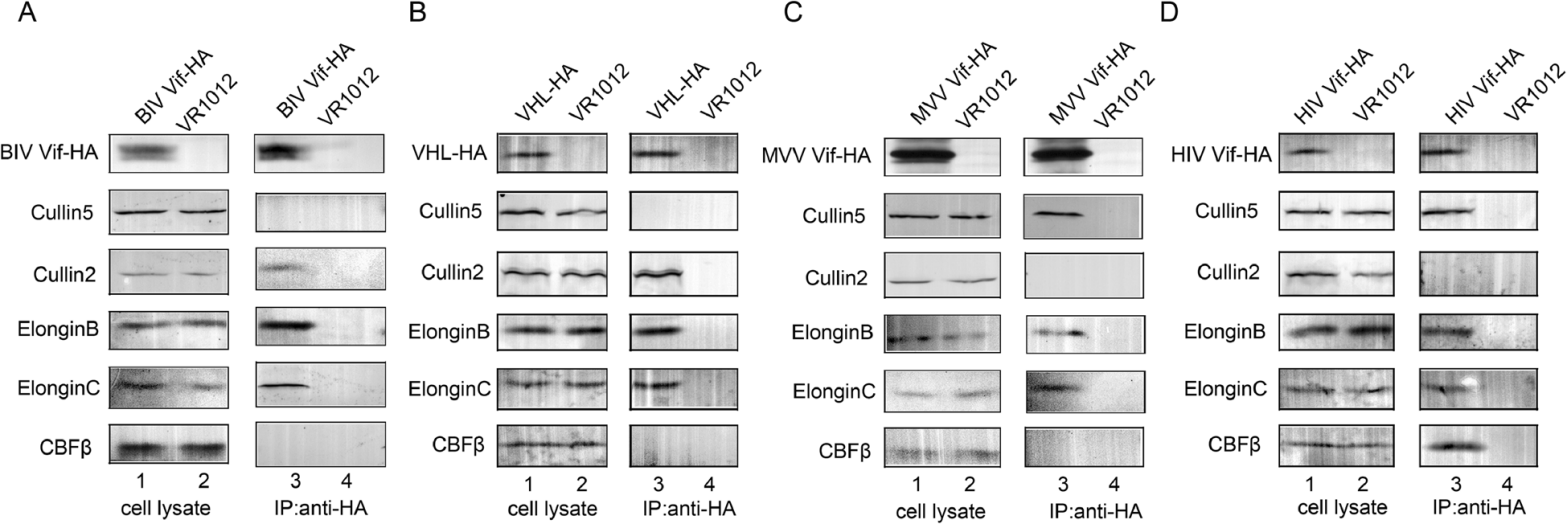
**BIV Vif recruits Cul2-EloB-EloC and MVV Vif recruits Cul5-EloB-EloC to induce btA3Z2-Z3 and oaA3Z2-Z3 degradation.** 293 T cells (5 × 10^6^) were transfected with 10 μg HA-tagged BIV Vif or 10 μg VHL and 10 μg VR1012 as a positive and a negative control, respectively **(A, B)**; or with 10 μg HA-tagged MVV Vif or 10 μg HIV Vif and 10 μg VR1012 as a positive and a negative control, respectively **(C, D)**. After 48 h of transfection, cell lysates were immunoprecipitated with HA beads, followed by SDS-PAGE and immunoblot analysis using anti-HA, anti-hCul2, anti-hCul5, anti-hEloB, anti-hEloC and anti-hCBF-β antibodies. All immunoprecipitation experiments were repeated three times.

HIV-1 Vif interacts with human Cul5, EloB and EloC to form the E3 complex, which degrades human A3G. A recent study showed that CBF-β is involved in this degradation process as well [3,12-17]. Therefore, we wondered whether host molecules participating in the degradation of A3 proteins by BIV and MVV Vif are the same or similar to those for HIV-1 Vif. Initially, we investigated the endogenous proteins involved in the degradation process by transfecting HA-tagged BIV Vif or HA-tagged MVV Vif into MDBK or MDOK cells; however, we did not obtain clear and convincing results due to the low transfection efficiency in these cells. Amino acid sequence alignments showed that the homologies of EloB, EloC, Cul2, Cul5 and CBF-β between humans, cattle and sheep exceed 98.0% (Table 1). Therefore, we attempted to study this issue by transfecting HA-tagged BIV Vif and HA-tagged von Hippel-Lindau (VHL) tumor suppressor (as a positive control for binding with Cul2), HA-tagged MVV Vif and HA-tagged HIV Vif (as a positive control for binding with Cul5) or negative vector control VR1012 into 293 T cells to perform a co- immunoprecipitation assay. Cell lysates were immunoprecipitated with HA beads, followed by SDS-PAGE and immunoblot analysis using anti-HA, anti-hCul2 anti-hCul5, anti-hEloB, anti-hEloC and anti-hCBF-β antibodies. The experimental results showed that the BIV Vif protein was capable of binding to endogenous Cul2, EloB and EloC proteins, but not with Cul5 or CBF-β (Figure 2A). Notably, the negative control HA-tagged VHL did not bind with CBF-β (Figure 2B). Meanwhile, the MVV Vif protein was capable of binding to endogenous Cul5, EloB and EloC proteins, but not with Cul2 or CBF-β (Figure 2C). The co-immunoprecipitation assay showed that BIV Vif recruited Cul2, EloB and EloC, while MVV Vif recruited Cul5, EloB and EloC, to form the E3 complex to induce the degradation of btA3Z2-Z3 and oaA3Z2-Z3, respectively. Of the various Vif proteins, BIV Vif appears to be unique by recruiting Cul2, and CBF-β was not found to be involved in the degradation of A3Z2-Z3 mediated by either BIV Vif or MVV Vif.

**Table 1:** **Homology rates of EloB, EloC, Cul2, Cul5 and CBF-**β **between humans, cattle and sheep**

**Species**	**Protein (% identity of amino acids)**				
	**EloB**	**EloC**	**Cul2**	**Cul5**	**CBFβ**
*Homo sapiens/Bos taurus*	98.3	100.0	99.7	100.0	100.0
*Homo sapiens/Ovis aries*	98.3	100.0	98.5	100.0	100.0
*Bos taurus/Ovis aries*	100.0	100.0	98.3	100.0	100.0

### BIV Vif interacts with btCul2 and btEloC directly but not with btCBF-β, and MVV Vif interacts with oaCul5 and oaEloC directly but not with oaCBF-β

**Figure 3:**
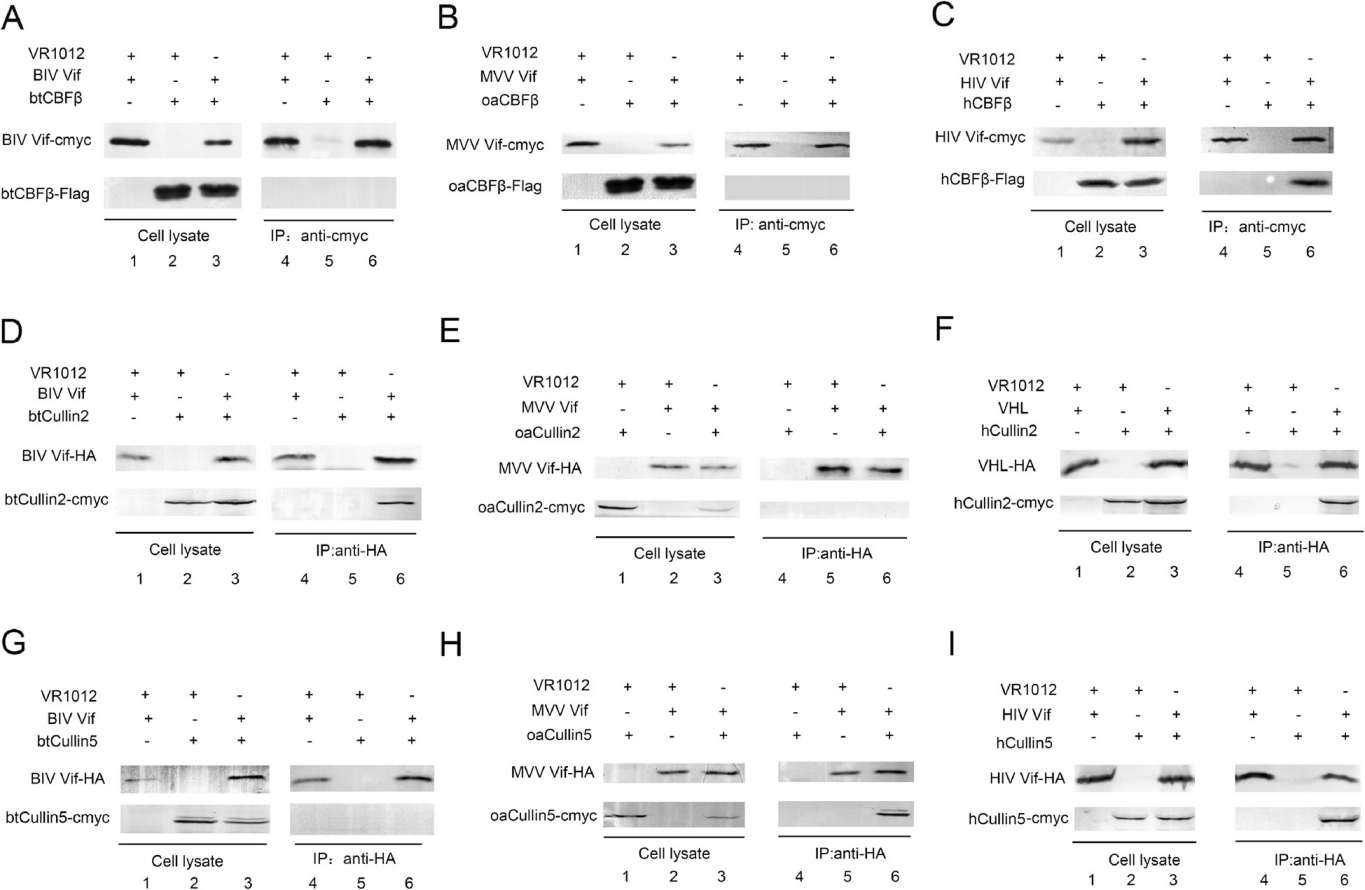
**BIV Vif interacts with btCul2 and MVV Vif with oaCul5, while CBF-β** **are not involved. (A–C)** 293 T cells (5 × 10^6^) were co-transfected with 5 μg cmyc-tagged BIV Vif (MVV Vif or HIV Vif) and 5 μg VR1012, 5 μg Flag-tagged btCBF-β (oaCBF-β or hCBF-β) and 5 μg VR1012, 5 μg cmyc-tagged BIV Vif (MVV Vif or HIV Vif) and 5 μg Flag-tagged btCBF-β (oaCBF-β or hCBF-β). At 48 h after transfection, cell lysates were co-immunoprecipitated with an anti-cmyc antibody, followed by SDS-PAGE and immunoblot analysis using anti-cmyc and anti-Flag antibodies. **(D-F)** 293 T cells (5 × 10^6^) were co-transfected with 6 μg HA-tagged BIV Vif (MVV Vif, or pVHL) and 10 μg VR1012, 10 μg cmyc-tagged btCul2 (oaCul2 or hCul2) and 6 μg VR1012, 6 μg HA-tagged BIV Vif (MVV Vif or pVHL) and 10 μg cmyc-tagged btCul2 (oaCul2 or hCul2). Cells were co-immunoprecipitated with HA beads followed by SDS-PAGE and immunoblot analysis using anti-HA and anti-cmyc antibodies. **(G-I)** 293 T cells (5 × 10^6^) were co-transfected with 4 μg HA-tagged BIV Vif (MVV Vif or HIV Vif) and 8 μg VR1012, 8 μg cmyc-tagged btCul5 (oaCul5 or hCul5) and 4 μg VR1012, 4 μg HA-tagged BIV Vif (MVV Vif or HIV Vif) and 8 μg cmyc-tagged btCul5 (oaCul5 or hCul5). Cells were co-immunoprecipitated with HA beads followed by SDS-PAGE and immunoblot analysis using an anti-HA and anti-cmyc antibodies. All co-immunoprecipitation experiments were repeated three times.

In order to further confirm the cellular proteins involved in BIV Vif-mediated degradation of btA3Z2-Z3, 293 T cells were transiently co-transfected with cmyc-tagged BIV Vif and Flag-tagged btCBF-β or HA-tagged btEloC (or HA-tagged BIV Vif and cmyc-tagged btCul2 or cmyc-tagged btCul5). Subsequently, co-immunoprecipitation experiments were performed to explore the interaction between BIV Vif and btCBF-β, btEloC, btCul2 and btCul5. After 48 h of transfection, cells were immunoprecipitated with HA beads or with an anti-cmyc antibody followed by SDS-PAGE and immunoblot analysis using an anti-HA antibody and an anti-cmyc antibody. The results revealed that BIV Vif could directly interact with btCul2, but not with btCul5 or btCBF**-**β (Figure 3A, D, G). BIV Vif could also bind with btEloC (data not shown). Of note, the btCBF-β-independent function of BIV Vif has been reported previously [14]. The same experiments were carried out as mentioned above for MVV Vif. The results revealed that MVV Vif could directly interact with oaCul5 and oaEloC, but not with oaCul2 or oaCBF-β (Figure 3B, E, H). MVV Vif could also interact with oaEloC (data not shown). 293 T cells were transiently co-transfected with cmyc-tagged HIV Vif and Flag-tagged hCBF-β (or HA-tagged HIV Vif and cmyc- tagged hCul5), HA-flagged hVHL and cmyc-tagged hCul2 as positive controls (Figure 3C, F, I).

### Mutation of BC box in BIV and MVV Vif or C-terminal hydrophilic residue replacement in btEloC and oaEloC can disrupt the activity of these Vif proteins against btA3Z2-Z3 and oaA3Z2-Z3, respectively

**Figure 4:**
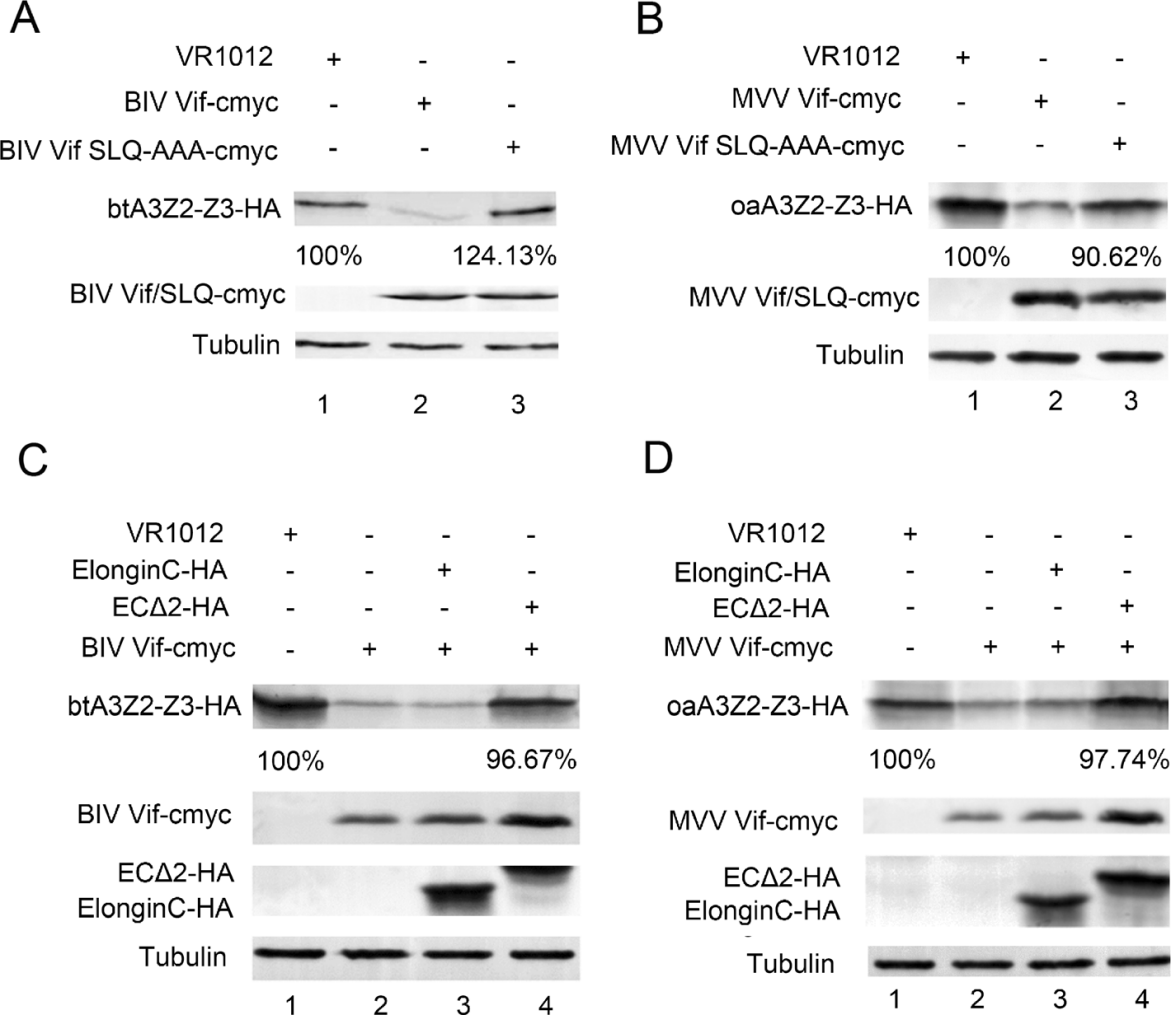
**Mutants of BIV/MVV Vif BC box and btEloC/oaEloC can disrupt the activity of BIV/MVV Vif. 293 T cells (0.5 × 10** ^**6**^ **) were co-transfected with (A) 30 ng HA-tagged btA3Z2-Z3 and 200 ng cmyc-tagged BIV Vif or BIV Vif SLQ-AAA, (B) 15 ng of HA-tagged oaA3Z2-Z3 and 200 ng cmyc-tagged MVV Vif or MVV Vif SLQ-AAA, (C) 30 ng HA-tagged btA3Z2-Z3 and 200 ng cmyc-tagged BIV Vif or VR1012, adjusted to 500 ng with 300 ng btEloC-HA, btEloC**Δ**2-HA or VR1012, (D) 15 ng HA-tagged oaA3Z2-Z3 and 200 ng cmyc-tagged MVV Vif or VR1012, adjusted to 500 ng with 300 ng of HA-tagged oaEloC, HA-tagged oaEloC**Δ**2 or VR1012.** At 48 h after transfection, the cells were harvested for Western blotting using anti-HA, anti-cmyc and anti-tubulin antibodies. All degradation experiments were repeated five times.

Lentiviral Vif proteins represent substrate receptor proteins that contain relatively conserved BC-box motifs. The known BC-box motif of HIV-1, SIVmac239, BIV and MVV Vifs is SLQ, and that of FIV Vif is TLQ [27,40]. In this study, we replaced the SLQ sequence of BIV Vif and MVV Vif with AAA in order to explore whether the BC box is critical for the degradation of btA3Z2-Z3 and oaA3Z2-Z3 induced by BIV Vif and MVV Vif, respectively. To investigate role of the SLQ motif in BIV Vif, 293 T cells were transfected with HA-tagged btA3Z2-Z3 and cmyc-tagged BIV Vif or cmyc-tagged BIV Vif SLQ-AAA. Likewise, the function of the SLQ motif in MMV Vif was examined by transfecting 293 T cells with HA-tagged oaA3Z2-Z3 and cmyc-tagged MVV Vif or cmyc-tagged MVV Vif SLQ-AAA. At 48 h after transfection, the cells were harvested for Western blotting using anti-HA, anti-cmyc and anti-tubulin antibodies. The results revealed that BIV Vif and MVV Vif SLQ-AAA respectively lost the ability to degrade btA3Z2-Z3 and oaA3Z2-Z3 (Figure 4A, lane 3; Figure 4B, lane 3), suggesting that the BC-box motif of each of these two Vif proteins is critical for the degradation of the corresponding A3Z2-Z3 target. These results are consistent with a previous report showing that the BIV Vif SLQ-AAA and MVV Vif SLQ-AAA mutants have altered function and fail to degrade btA3Z3 and oaA3Z2-Z3 proteins, respectively [40] .

To further confirm the significance of EloC in the E3 ligase, we constructed btEloC and oaEloC mutants by replacement of critical hydrophobic amino acids A100 and L103 with hydrophilic serine [41]. 293 T cells were transfected with HA-tagged btA3Z2-Z3 and cmyc-tagged BIV Vif or VR1012 with HA-tagged btEloCΔ2 or HA-tagged btEloC as a control. In a parallel experiment, 293 T cells were transfected with HA-tagged oaA3Z2-Z3 and cmyc-tagged MVV Vif or VR1012 with HA-tagged oaEloCΔ2 or HA-tagged oaEloC as a control. At 48 h after transfection, the cells were harvested for Western blotting using anti-HA, anti-cmyc and anti-tubulin antibodies. Addition of EloCΔ2 rescued the levels of both btA3Z2-Z3 and oaA3Z2-Z3 (Figure 4C, lane 4; Figure 4D, lane 4). These results indicated that EloC takes part in the degradation of btA3Z2-Z3 by BIV Vif and of oaA3Z2-Z3 by MVV Vif.

### Dominant-negative btCul2 and oaCul5 mutants can inhibit the activity of BIV Vif against btA3Z2-Z3 and MVV Vif against oaA3Z2-Z3,respectively, and Zn is important for BIV Vif activity

**Figure 5:**
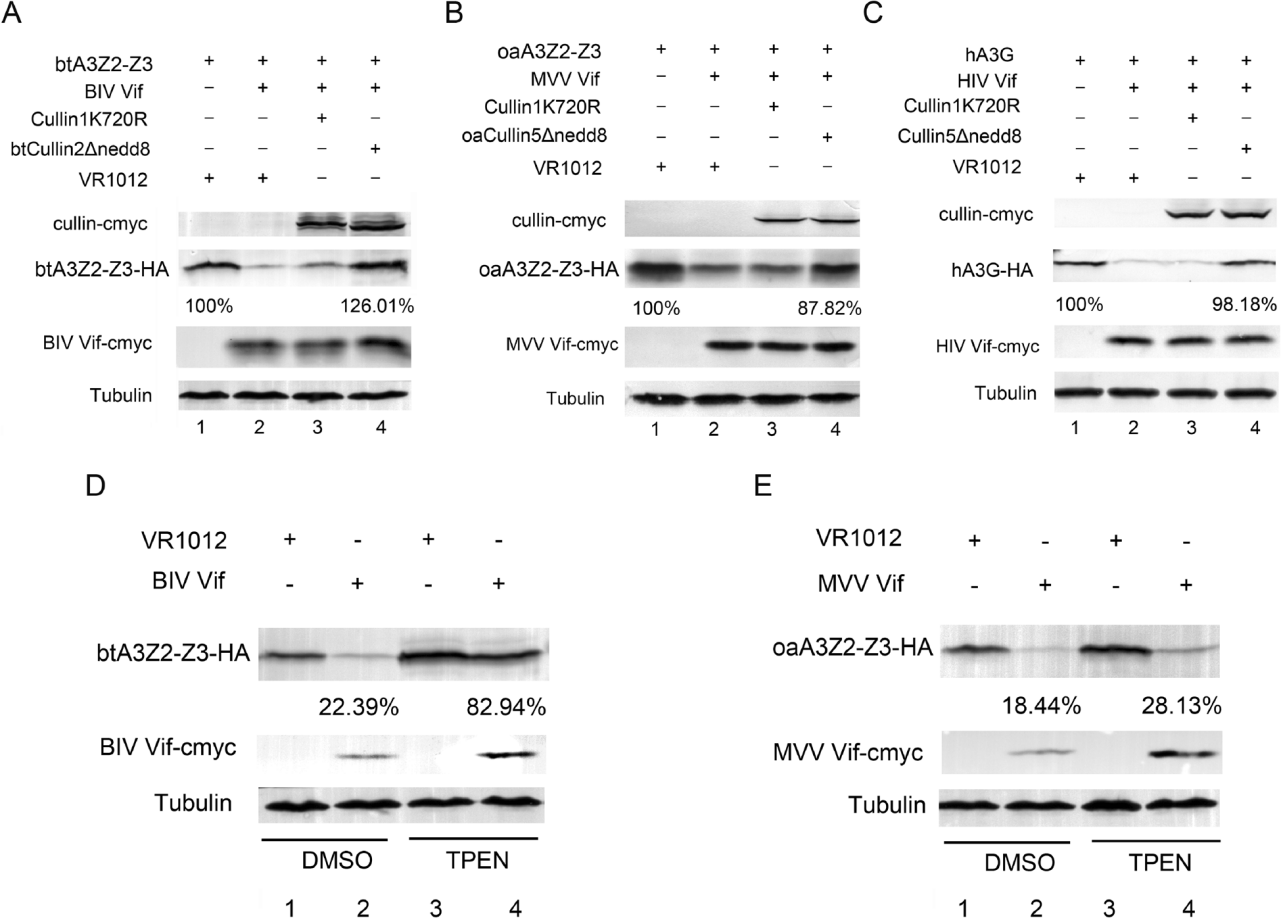
**Dominant-negative btCul2 and oaCul5 mutants block the degradation, while TPEN can inhibit btA3Z2-Z3 degradation.** 293 T cells (0.5 × 10^6^) were co-transfected with **(A)** 30 ng of HA-tagged btA3Z2-Z3 and 200 ng cmyc-tagged BIV Vif or VR1012, adjusted to 500 ng with 300 ng cmyc-tagged btCul2ΔNedd8, a control vector cmyc-tagged hCul1K720R or VR1012, **(B)** 15 ng HA-tagged oaA3Z2-Z3 and 200 ng cmyc-tagged MVV Vif or VR1012, adjusted to 500 ng with 300 ng cmyc-tagged oaCul5ΔNedd8, a control vector cmyc-tagged hCul1K720R or VR1012, **(C)** 50 ng HA-tagged hA3G and 600 ng cmyc-tagged HIV Vif or VR1012, adjusted to 900 ng with 300 ng cmyc-tagged btCul5ΔNedd8, a control vector cmyc-tagged hCul1K720R or VR1012. At 48 h after transfection, the cells were harvested for Western blotting using anti-HA, anti-cmyc and anti-tubulin antibodies. The percentages of btA3Z2-Z3, oaA3Z2-Z3 or hA3G in the presence of btCul2ΔNedd8, oaCul5ΔNedd8 or hCul5ΔNedd8 relative to that in the absence of BIV/MVV/HIV Vif (set to 100%) were calculated. 293 T cells (0.5 × 10^6^) were co-transfected with **(D)** 30 ng HA-tagged btA3Z2-Z3 and 200 ng cmyc-tagged BIV Vif or VR1012, **(E)** 15 ng HA-tagged oaA3Z2-Z3 and 200 ng cmyc-tagged MVV Vif or VR1012. After 36 h of transfection, the cells were treated with TPEN at 3.5 μM or DMSO as a control. At 48 h after transfection, the cells were harvested for Western blotting using anti-HA, anti-cmyc and anti-tubulin antibodies. Percentages of degradation with DMSO or TPEN treatment were calculated.

All Cullin family members are known to be modulated by the ubiquitin-like small molecule Nedd8, which is critical for E3 ubiquitin ligase activity [42]. To further confirm the participation of btCul2 and oaCul5 in the degradation of btA3Z2-Z3 and oaA3Z2-Z3, we constructed dominant-negative btCul2 and oaCul5 mutants and determined their effect on the degradation of btA3Z2-Z3 and oaA3Z2-Z3 induced by BIV and MVV Vif, respectively. 293 T cells were transfected with HA-tagged btA3Z2-Z3 and cmyc-tagged BIV Vif or VR1012, with cmyc-tagged btCul2ΔNedd8 or with a control vector expressing cmyc-tagged hCul1K720R, which is a dominant-negative hCul1 mutant [43]. In parallel, another set of 293 T cells was transfected with HA-tagged oaA3Z2-Z3 and cmyc-tagged MVV Vif or VR1012, with cmyc-tagged oaCul5ΔNedd8 or with the cmyc-tagged hCul1K720R control vector. As a positive control for the downregulation of hA3G by hCul5ΔNedd8, 293 T cells were transfected with HA-tagged hA3G and cmyc-tagged HIV Vif or VR1012, with cmyc-tagged hCul5ΔNedd8 or the cmyc-tagged hCul1K720R control vector. At 48 h after transfection, the cells were harvested for Western blotting using anti-HA, anti-cmyc and anti-tubulin antibodies. As expected, the expression of btCul2ΔNedd8 and oaCul5ΔNedd8 could up-regulate the amount of btA3Z2-Z3 and oaA3Z2-Z3, respectively (Figure 5A, lane 4; Figure 5B, lane 4). The results indicated that btCul2 and oaCul5 are recruited to the Cul-E3 complex.

Cellular proteins assemble with Cul-EloB-EloC E3 complexes through a BC box and a downstream Cul box [44]. Some cellular proteins such as the tumor suppressor VHL use a Cul2 box to bind with Cul2, while others such as SOCS3 use a Cul5 box to bind with Cul5 [44,45]. Primate lentiviral (HIV-1/SIV) Vif proteins use a zinc-binding HCCH motif to interact with Cul5 [25]. FIV Vif has neither a Cul5 box nor an HCCH motif, but it still interacts with Cul5 in a novel fashion [31]. Since BIV and MVV Vif have no apparent Cul2 or Cul5 box or an HCCH motif, we wondered whether Zn is significant for its interaction with btCul2 and oaCul5. In order to explore this issue, we used the membrane-permeable zinc chelator TPEN [46] to determine its effects on the BIV and MVV Vif-mediated degradation of btA3Z2-Z3 and oaA3Z2-Z3, respectively. 293 T cells were transfected with HA-tagged btA3Z2-Z3 and cmyc-tagged BIV Vif or VR1012. A parallel set of 293 T cells was transfected with HA-tagged oaA3Z2-Z3 and cmyc-tagged MVV Vif or VR1012. After 36 h of transfection, the cells were treated with TPEN at 3.5 μM (Figure 5D, lanes 3, 4; Figure 5E, lanes 3, 4) or DMSO (Figure 5D, lanes 1, 2; Figure 5E, lanes 1, 2). At 48 h after transfection, the cells were harvested for Western blotting using anti-HA, anti-cmyc and anti-tubulin antibodies. The results showed that the addition of TPEN blocked the degradation of btA3Z2-Z3 induced by BIV Vif (Figure 5D, lane 4), but it had a minimal effect on the degradation of oaA3Z2-Z3 induced by MVV Vif (Figure 5E, lane 4). These findings indicated that Zn is important for the activity of BIV Vif, but it is not required for the activity of MVV Vif. We propose that a zinc finger domain is involved in the BIV Vif-mediated degradation of btA3Z2-Z3, while MVV Vif may have a yet undefined mechanism for interacting with oaCul5, which is similar to FIV Vif.

### CCHC motif is crucial for activity of BIV Vif and its interaction with Cul2

**Figure 6:**
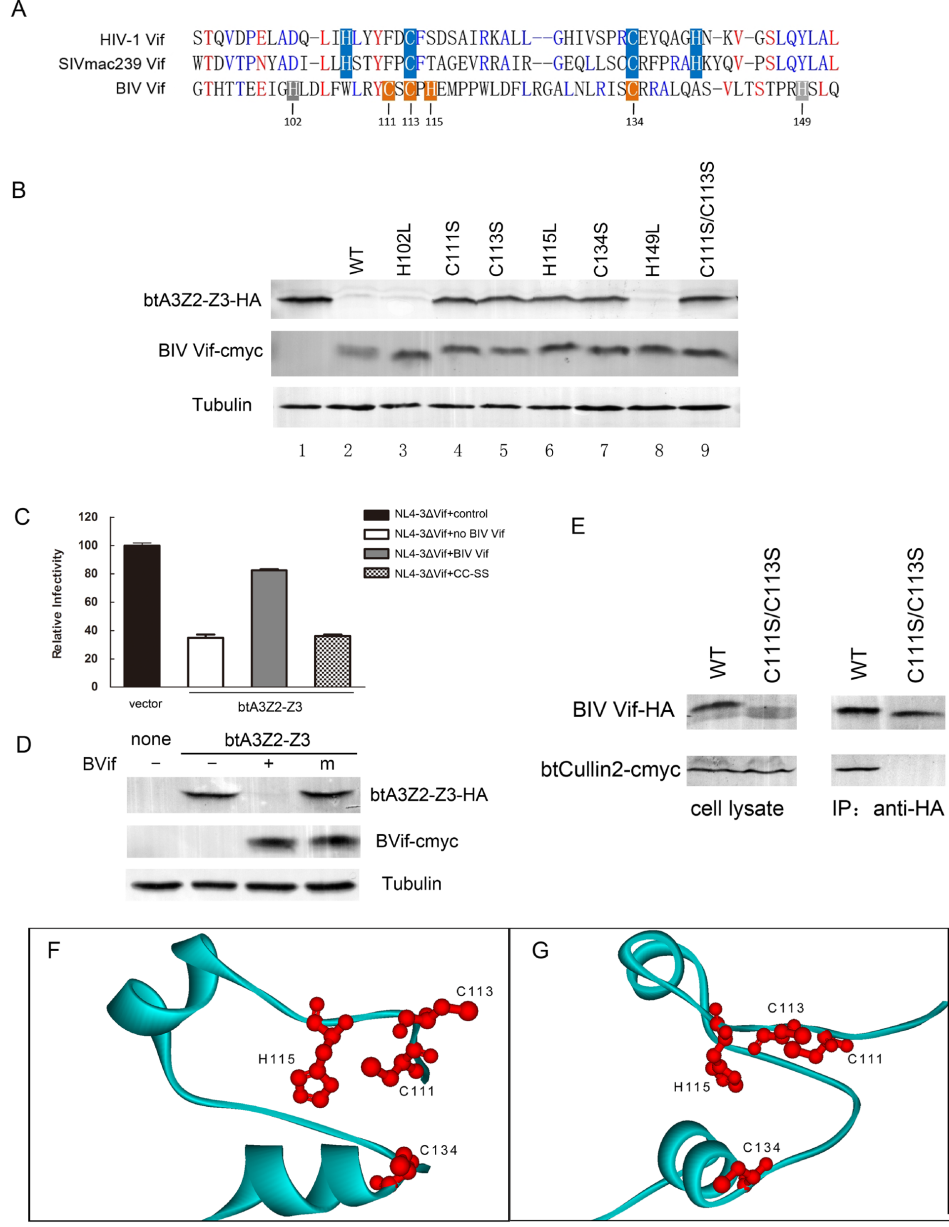
**The C-x1-C-x1-H-x19-C motif is crucial and predicted to be a zinc binding loop. (A)** Alignment of partial BIV Vif sequences with primate lentiviral Vifs by BioEdit. **(B)** 293 T cells (0.5 × 10^6^) were co-transfected with 30 ng HA-tagged btA3Z2-Z3 and 200 ng cmyc-tagged BIV Vif or BIV Vif mutants H102L, C111S, C113S, H115L, C134S, H149L or C111S/C113S. At 48 h after transfection, the cells were harvested for Western blotting using anti-HA, anti-cmyc and anti-tubulin antibodies. (**C** and **D**) 293 T cells (1 × 10^6^) were co-transfected with 1 μg pNL4-3ΔVif plus 15 ng VR1012, or HA-tagged btA3Z2-Z3 and 100 ng cmyc-tagged BIV Vif, BIV Vif C111S/C113S or VR1012. The virus infectivity was assayed by the MAGI assay. Virus infectivity was set to 100% in the absence of btA3Z2-Z3. **(D)** Western blot was performed on the cell lysates from **(C)** to show the producer cell levels of btA3Z2-Z3 protein (anti-HA), BIV Vif/BIV Vif C111S/C113 (anti-cmyc) and tubulin. **(E)** 293 T cells (5 × 10^6^) were co-transfected with 10 μg cmyc-tagged btCul2 and 6 μg HA-tagged BIV Vif or 6 μg BIV Vif C111S/C113S. At 48 h after transfection, cells were immunoprecipitated with HA beads, followed by SDS-PAGE and immunoblot analysis using an anti-HA antibody and an anti-cmyc antibody. **(F** and **G)** Panels A and B are views of the alpha carbon ribbon and differ from each other by 90 degrees. Residues which likely participate in the coordination of zinc ions are shown and labeled. All infection experiments were repeated three times.

In order to further explore the mechanism of the interaction between BIV Vif and btCul2, we conducted an in-depth analysis of the BIV Vif sequence. A putative zinc binding motif H-x_8_-C-x_1_-C-x_1_-H-x_19_-C-x_14_-H was found upstream of the BC box. To explore whether this putative motif is a functional domain for the activity of BIV Vif, we constructed a series of BIV Vif single mutants in which histidines and cysteines (H102, C111, C113, H115, C134, and H149) were replaced individually with leucine or serine and a BIV Vif double mutant containing two amino acids (C111 and C113) replaced with serine. 293 T cells were co-transfected with HA-tagged btA3Z2-Z3 and cmyc-tagged BIV Vif, cmyc-tagged BIV Vif H102L, cmyc-tagged BIV Vif C111S, cmyc-tagged BIV Vif C113S, cmyc-tagged BIV Vif H115L, cmyc-tagged BIV Vif C134S, cmyc-tagged BIV Vif H149L or cmyc-tagged BIV Vif C111S/C113S. At 48 h after transfection, the cells were harvested for Western blotting using anti-HA, anti-cmyc and anti-tubulin antibodies. The results revealed that BIV Vif C111S, BIV Vif C113S, BIV Vif H115L and BIV Vif C134S lost almost all of their abilities to degrade btA3Z2-Z3 (Figure 6B, lanes 4, 5, 6, 7, 9), implying that the C-x_1_-C-x_1_-H-x_19_-C motif is critical for the degradation activity of BIV Vif. The BIV Vif CCHC mutant also lost the ability to suppress the antiviral activity of btA3Z2-Z3 (Figure 6C, D), supporting the hypothesis that the CCHC motif of BIV Vif is critical for its activity against btA3Z2-Z3. To further confirm whether the CCHC motif is a critical domain for interacting with Cul2, 293 T cells were transiently transfected with cmyc-tagged btCul2 and VR-BIV Vif C111S/C113S-HA. BIV Vif was used as a positive control. Subsequently, co-immunoprecipitation experiments were performed to explore the interaction between btCul2 and BIV Vif or BIV Vif C111S/ C113S. After 48 h of transfection, cells were immunoprecipitated with HA beads or with an anti-cmyc antibody, followed by SDS-PAGE and immunoblot analysis using an anti-HA antibody and an anti-cmyc antibody. The results revealed that double mutations in the CCHC motif of BIV Vif could completely block the interaction between BIV Vif and btCul2 (Figure 6E). However, this CCHC motif is different from all previously reported zinc finger structures (46), including the HCCH zinc finger in primate lentiviral Vifs. To further explore whether it is a zinc finger, we built a homology model for this potential zinc binding domain of BIV Vif (Figure 6F, G). The model implies this motif should be able to form a proper zinc coordination site. Although this motif is unlike all previously reported zinc fingers, the sequence and potential zinc coordinated motif of this domain are similar to those of “zinc binding loops”, as described previously [47].

## Discussion

### Mechanisms of BIV Vif- and MVV Vif-induced degradation of btA3Z2-Z3 and oaA3Z2-Z3 differ by the respective utilization of Cul2 and Cul5

Based on numerous investigations, HIV-1 Vif is known to recruit the transcription cofactor CBF-β and EloB-EloC to the Cul5-Rbx complex, thereby forming the E3 ubiquitin ligase and inducing the degradation of human antiviral proteins [12-17,28,48]. Other studies have explored further the degradation mechanisms of SIV Vif against RhA3G and FIV Vif against feline A3s [29-31]. In the current study, we investigated the effects of BIV and MVV Vifs on the stability of btA3Z2-Z3 and oaA3Z2-Z3, respectively. First, we found that the proteasome inhibitor MG132 [39] could block the degradation effect of BIV Vif on btA3Z2-Z3 and that of MVV Vif on oaA3Z2-Z3, suggesting that they are proteasome-dependent processes. In addition, BIV and MVV Vifs were found to affect the synthesis rate rather than the stability of btA3Z2-Z3 and oaA3Z2-Z3, respectively. The endogenous immunoprecipitation experiments showed that btEloB, btEloC and btCul2 are involved in the degradation of btA3Z2-Z3 by BIV Vif. Correspondingly, oaEloB, oaEloC and oaCul5 are involved in the degradation of oaA3Z2-Z3 by MVV Vif. Meanwhile, the host CBF-β proteins do not play a role in this function of either BIV or MVV Vif. Co-immunoprecipitation assays further verified the direct interaction of BIV Vif with btEloC and btCul2, but not with btCBF-β or btCul5. MVV Vif also was confirmed to directly interact with oaEloC and oaCul5, but not with oaCBF-β or oaCul2. Similarly, we found no interaction between FIV Vif and feline CBF-β (data not shown). CBF-β is reported to regulate Vif-Cul5 ligase by promoting the folding of primate lentiviral Vifs (HIV-1 and SIV) [14,29,48,49]. The fact that other mammalian Vifs do not require the participation of CBF-β for their function implies that BIV/MVV/FIV Vif may utilize a different mechanism for recruiting the Cul-E3 ligase or factors other than CBF-β which have not been discovered in the degradation of btA3Z2-Z3 by BIV Vif and that of oaA3Z2-Z3 by MVV Vif.

We constructed an EloC mutant in which the key hydrophobic amino acids (A100 and L103) were substituted with hydrophilic serine, which disrupted the interaction between the EloC and the BC box of the cellular proteins [41]. These mutations disrupted the degradation of btA3Z2-Z3 induced by BIV Vif and that of oaA3Z2-Z3 induced by MVV Vif, implicating the involvement of EloC in the process. Although the sequence homology of lentiviral Vifs is poor, they share a highly conserved S/TLQY/RLA motif, which is a BC box essential for the binding of EloC [12,27,28]. The SLQ mutations in BIV and MVV Vifs could block the degradation of btA3Z2-Z3 and oaA3Z2-Z3, confirming that the BC-box motifs in these Vifs are essential for the recruitment of the E3 complex. These two experiments both showed that EloC is a member of the E3 ligase complex. The participation of btCul2 in the degradation induced by BIV Vif and that of oaCul5 in the degradation induced by MVV Vif was further verified by use of dominant-negative btCul2 and oaCul5 mutants. The disruption of BIV Vif-induced degradation of btA3Z2-Z3 and MVV Vif-induced degradation of oaA3Z2-Z3 by the corresponding mutants demonstrated that btCul2 and oaCul5 are required for the BIV and MVV Vif activity against btA3Z2-Z3 and oaA3Z2-Z3, respectively.

### CCHC motif is crucial for BIV Vif activity and predicted to be a zinc binding loop

Primate lentiviral Vifs contain a zinc coordination site H-x_5_-C-x_17– 18_-C-x_3 –5_-H (HCCH) [24-26,48], which determines the selective recruitment of Cul5 by HIV-1 and SIV Vif. Although FIV Vif does not contain an HCCH domain, it can still recruit Cul5 [31]. Previous research has indicated that FIV Vif may utilize a new non-zinc finger dependent mechanism for interacting with Cul5 [31]. These findings suggest that the Cul2 box and Cul5 box downstream of the BC box or HCCH domain are not absolutely necessary for the recruitment of Cul2 or Cul5. In this study, we found a potential zinc finger, the C-x_1_-C-x_1_-H-x_19_-C motif, which was critical for the degradation activity of BIV Vif and the interaction with a Cul protein. This motif is different from all previously reported zinc finger structures, including the HCCH zinc finger in primate lentiviral Vifs, but the sequence and potential zinc coordinated motif of this domain are almost identical to those of “zinc binding loops” (45), except the distance between the 3rd (H115) and 4th (C134) residues is much longer in our protein. We then further analyzed the sequence of this potential zinc binding domain and found only one hit covering the full-length sequence of this domain, the crystal structure of tatD DNase of ***Escherichia coli***, which is also a metallic ion binding protein [50]. Using this structure as a template, we built a homology model for the potential zinc binding domain of BIV Vif. The model suggests that the entire domain likely forms an alpha-beta-alpha super secondary structure. The 4th residue of this potential zinc domain folds back through this structure and is distally close to the other three residues. Thus, they should be able to form a proper zinc coordination site, which is also consistent with our mutagenesis studies.

### Various mechanisms of lentiviral Vif-mediated degradation of A3 proteins are slightly different

Slight differences in various mechanisms of lentiviral Vif-mediated degradation of A3 proteins (Table 2) may be attributed to different factors involved. First, the host proteins involved in the degradation are different. Primate lentiviral Vifs require CBF-β as a regulator of the folding of Vif to neutralize the A3 proteins, but non-primate lentiviral Vifs, including those of FIV, BIV and MVV, do not require CBF-β to neutralize the A3 proteins. All lentiviral Vifs recruit Cul5 except for BIV Vif, which was found here to recruit Cul2. Second, the mechanisms of interaction between the Vif protein and Cul protein are different. Primate lentiviral Vifs utilize a highly conserved HCCH zinc-binding motif to bind with Cul5. By contrast, BIV Vif may utilize the CCHC domain to bind with Cul2, while FIV and MVV may use a novel method for recruiting Cul5.

**Table 2:** **Comparison of various mechanisms of lentiviral Vif-mediated degradation of A3 proteins**

**Lentivirus**	**E3 complex**	**Method of binding with EloC**	**Cul2/Cul5**	**Method of binding with Cul**	**CBF-β** **involved**
**HIV**	**Y**	**BC box (SLQ)**	**Cul5**	**zinc finger**	**Y**
**SIV**	**Y**	**BC box (SLQ)**	**Cul5**	**zinc finger**	**Y**
**FIV**	**Y**	**BC box (TLQ)**	**Cul5**	**unknown**	**N**
**BIV**	**Y**	**BC box (SLQ)**	**Cul2**	**zinc binding loop**	**N**
**MVV**	**Y**	**BC box (SLQ)**	**Cul5**	**unknown**	**N**

## Conclusions

Overall, this study supplements our knowledge of the mechanism of degradation of host antiviral proteins induced by BIV Vif and MVV Vif. Our work described the interaction between BIV Vif and btCul2 through a previously unreported zinc binding loop [C-x_1_-C-x_1_-H-x_19_-C] which may provide a foundation for further studies on similar protein-protein interactions. The CBF-β-independent degradation pathway suggests that the degradation of btA3Z2-Z3 by BIV Vif and that of oaA3Z2-Z3 by MVV Vif may require factors different from CBF-β. These viruses and their hosts have co-evolved various mutually antagonistic proteins, which over the long evolutionary process have facilitated viral entry into new hosts. Thus, our work may shed light on the course of disease in cows and sheep, as well as the potential for cross-species transmission of BIV or MVV. Further studies to identify these factors may provide new insights into the molecular mechanism(s) of Vif-mediated neutralization of host innate immune defenses.

## Methods

### Plasmid construction

The genes encoding btEloB, btEloC, btCBF-β, btCul2, btCul5, oaEloB, oaEloC, oaCBF-β, oaCul2 and oaCul5 were obtained by RT-PCR. Bovine total RNA was extracted from MDBK cells, and ovine total RNA was extracted from MDOK cells using TRIzol (Invitrogen, Carlsbad, CA) separately. The following primers were used to amplify the genes by RT-PCR: btEloB, forward (5′-ATGGACGTGTTCCT CATGATC-3′) and reverse (5′-TCACTGCACAGCCT GTTCGTTG-3′); btEloC, forward (5′-ATGGATGGAGAAGAGAAAAC-3′) and reverse (5′-TTAACAATCTA GGAAGTTC-3′); btCBF-β, forward (5′-ATGCCGCGCGTCGTGCCCGAC-3′) and reverse (5′-CTAGGGTCTTGTTGTCTTCTT-3′); btCul2, forward (5′-ACAC TAAACTTGCACAATGTCTTT-3′) and reverse (5′-TCAGGCGACGTAGCTGTAC TCATCT-3′); btCul5, forward (5′-GAGTCTAAGTTGAAGGAACATG-3′) and reverse (5′-ATTGTCCATGATATTCAAAATTA-3′); oaEloB, forward (5′-ATGGACG TGTTCCTCATGAT-3′) and reverse (5′-TCACTGCACAGCCTGTTCGT-3′); oaEloC, forward (5′-ATGGATGGAGAAGAGAAAAC-3′) and reverse (5′-TTAACA ATCTAGGAAGTTTG-3′); oaCBF-β, forward (5′-ATGCCGCGCGTCGTGCCC GAC-3′) and reverse (5′-CTAGGGTCTTGTTGTCTTCTT-3′); oaCul2, forward (5′-ACACTAAACTTGCACAATGTCTTT-3′) and reverse (5′-TCAGGCGACGTAG CTGTACTCATCT-3′); and oaCul5, forward (5′-ATGGCGACGTCTAA TCTGTT-3′) and reverse (5′-TTACGCCATATATATGAAAG-3′). The amplified genes were inserted into the intermediate pGEM-T-easy vector (Promega, Madison, WI) to generate bt- and oaEB-T-easy, bt- and oaEC-T-easy, bt- and oaCBF-β-T-easy, bt- and oa Cul2-T-easy and bt- and oaCul5-T-easy.

The btA3Z2-Z3 and oaA3Z2-Z3 eukaryotic expression plasmids were derived from BtA3Z2-Z3 and OaA3Z2-Z3 expression constructs described previously [37], and PC-hVHL-HA was provided by Xiao-Fang Yu (Johns Hopkins University, Baltimore, MD). The lentiviral Vifs chosen for codon optimization (Sangon Biotech, Shanghai, China) matched BIV_BIM127_ (gb M32690) and MVV Icelandic strain 1514 (gb M60610). The VR-btELoB-cmyc, btCul2-cmyc and btCul5-cmyc plasmids were obtained by PCR amplification from the btELoB-T-easy, bt-Cul2-T-easy and btCul5-T-easy plasmids separately and subcloned into the VR1012 vector for eukaryotic expression with a cmyc tag added at the C-terminus. The VR-btELoC-HA plasmid was obtained by PCR amplification from btELoC-T-easy and subcloned into the VR1012 vector for eukaryotic expression with a HA tag added at the C-terminus. The VR-btCBF-β-flag plasmid was obtained by PCR amplification from btCBF-β-T-easy and subcloned into the VR1012 vector for eukaryotic expression with a Flag tag added at the C-terminus. The VR-oaELoB-cmyc, oaCul2-cmyc and oaCul5-cmyc plasmids were obtained by PCR amplification from the oaELoB-T-easy, oaCul2-T-easy and oaCul5-T-easy plasmids separately and subcloned into the VR1012 vector for eukaryotic expression with a cmyc tag added at the C-terminus. The VR-oaELoC-HA plasmid was obtained by PCR amplification from oaELoC-T-easy and subcloned into the VR1012 vector for eukaryotic expression with a HA tag added at the C-terminus. The VR-oaCBF-β-flag plasmid was obtained by PCR amplification from oaCBF-β-T-easy and subcloned into the VR1012 vector for eukaryotic expression with a Flag tag added at the C-terminus.

VR-BIV Vif-cmyc is a eukaryotic plasmid expressing the codon-optimized BIV_BIM127_ (gb M32690) Vif. It was generated by adding a cmyc tag to the C-terminus of the codon-optimized BIV *vif* gene and then subcloned into the VR1012 vector at the *Bam*HI and *Not*I restriction sites. VR-BIV Vif -HA was derived from VR-BIV Vif-cmyc with primers that added an HA tag in frame at its C-terminus. VR-btCul2-HA, and VR-btCul5-HA were similarly obtained from corresponding cmyc-tagged plasmids (VR-btCul2-cmyc and VR-btCul5-cmyc, respectively).

Via site-directed mutagenesis, VR-BIV Vif SLQ-AAA-cmyc, VR-BIV Vif H102L-cmyc, VR-BIV Vif C111S-cmyc, VR-BIV Vif C113S-cmyc, VR-BIV Vif H115L-cmyc, VR- BIV Vif C134S-cmyc, VR-BIV Vif H149L-cmyc and VR-BIV Vif C111S/ C113S-cmyc were derived from VR-BIV Vif-cmyc. Meanwhile, VR-BIV Vif C111S/C113S-HA was derived from VR-BIV Vif-HA. The btCul2, btCul5 and btEloC mutants were engineered based on the corresponding plasmids (VR-btCul2-cmyc, VR-btCul5-cmyc and VR-btEloC-HA, respectively). These mutant constructs were made using the QuickChange mutagenesis system (Stratagene, La Jolla, CA) and confirmed by sequencing.

VR-MVV Vif-cmyc is a eukaryotic plasmid expressing the codon-optimized MVV Icelandic strain 1514 *vif*. It was generated by adding a cmyc tag to the C-terminus of the codon-optimized MVV *vif* gene and then subcloned into the VR1012 vector at the *Sal*I and *Not*I restriction sites.

VR-MVV Vif SLQ-AAA-cmyc was derived from VR-MVV Vif-cmyc via site-directed mutagenesis, and the oaCul2, oaCul5 and oaEloC mutants were engineered based on the corresponding plasmids (VR-oaCul2-cmyc, VR-oaCul5-cmyc and VR-oaEloC-HA, respectively). VR1012 clone using the QuickChange mutagenesis system and confirmed by sequencing.

The expression vectors VR-HIV Vif-cmyc, PC-hA3G–HA, VR-hEC-HA, VR- hCBF-β-Flag and the infectious molecular clone pNL4-3ΔVif were described previously [12,16,51]. The expression vectors VR-hCul5-cmyc, VR-hCul5 ΔNedd8-cmyc and VR-hCul1K720R-cmyc were derived from VR-hCul5-HA, VR-hCul5 ΔNedd8-HA and VR-hCul1K720R-HA, respectively, as described previously [43,52].

### Cells and transfections

HEK293T (CRL-11268) cells, MDBK (CCL-22) cells and MDOK (CRL-1633) cells were purchased from the American Type Culture Collection (ATCC, Manassas, VA). MAGI-CCR5 cells (catalog number 3522) were obtained from the NIH AIDS Research and Reference Reagent Program (NIH-ARRRP). HEK293T and MAGI-CCR5 cells were cultured in Dulbecco’s Modified Eagle’s Medium (DMEM) supplemented with 10% fetal bovine serum (FBS) at 37°C with 5% CO_2_. MDBK cells were cultured in DMEM supplemented with 10% horse serum at 37°C with 5% CO_2_. MDOK cells were cultured in Minimum Essential Medium (MEM) supplemented with 10% FBS at 37°C with 5% CO_2_. Transfections of 293 T cells were performed using Lipofectamine 2000 (Invitrogen) according to the manufacturer’s instructions.

### Antibodies

The following antibodies were used in the present study: anti-HA mouse monoclonal antibody (mAb; Covance, Emeryville, CA), anti-cmyc mouse mAb (Millipore, Billerica, MA), anti-Flag mouse mAb (Sigma, St. Louis, MO), anti-tubulin mouse mAb (Covance), anti-Cul2 rabbit polyclonal antibody (pAb; Santa Cruz Biotechnology, Santa Cruz, CA), anti-Cul5 rabbit pAb (Santa Cruz Biotechnology), anti-CBF-β mouse mAb (Santa Cruz Biotechnology), anti-EloB rabbit pAb (Santa Cruz Biotechnology) and anti-EloC mouse mAb (Santa Cruz Biotechnology).

### Western blotting

Cells were collected 48 h post-transfection and lysed with sodium dodecyl sulfate (SDS) sample buffer. The samples were boiled for 20 min and separated by SDS-polyacrylamide gel electrophoresis (PAGE) and then transferred onto nitrocellulose membranes (Whatman, Kent, UK). After blocking in 5% nonfat milk, the membranes were probed with various primary antibodies against proteins of interest. Secondary antibodies were alkaline phosphatase-conjugated anti-rabbit, anti-mouse IgG (Jackson Immunoresearch, West Grove, PA). Immunoreactions were detected with 5-bromo-4-chloro-3-indolylphosphate (BCIP) and nitro blue tetrazolium chloride (NBT) solutions.

### MG132 inhibition assay

To determine if the degradation of btA3Z2-Z3 by BIV Vif or that of oaA3Z2-Z3 by MVV Vif is proteasome-dependent, 293 T cells were treated with the proteasome inhibitor MG132 (Sigma–Aldrich) at 10 μM and DMSO as negative control at 36 h after transfection with indicated plasmids. At 48 h after transfection, 293 T cells were harvested and analyzed by Western blotting.

### TPEN inhibition assay

To explore if Zn is significant for the degradation of A3Z2-Z3 induced by Vif, 293 T cells were treated with TPEN at 3.5 μM and DMSO as negative control at 36 h after transfection with indicated plasmids. At 48 h after transfection, 293 T cells were harvested and analyzed by Western blotting.

### CHX-treated A3Z2-Z3 stability assay

At 36 h after transfection with indicated plasmids, 293 T cells were treated with CHX (Sigma–Aldrich) at the final concentration of 100 μg/ml for 0, 6, 12, 24 h and then harvested and analyzed by Western blotting.

### Immunoprecipitation assay

At 48 h after transfection with indicated plasmids, 293 T cells were obtained and dissociated in lysis buffer (50 mM Tris, pH 7.5, with 150 mM NaCl, 1% Triton X-100 and complete protease inhibitor cocktail tablets) at 4°C for 1 h, followed by centrifugation at 10,000 × *g* for 10 min at 4°C to pellet the cell debris. The pre-cleared supernatants were collected and then mix with anti-HA Ab-conjugated agarose beads (Roche, Mannheim, Germany), followed by incubation at 4°C for 3 h. Alternatively, the pre-cleared supernatants were collected and incubated with mouse anti-cmyc (Millipore) for 1 h and then mix with Protein G-agarose (Roche), followed by incubation at 4°C for 3 h. The beads were washed three times with wash buffer (20 mM Tris, pH 7.5, with 100 mM NaCl, 0.1 mM EDTA and 0.05% Tween 20), and the pellet was resuspended in 30 μl glycine HCl (pH 2.0) elution buffer. The eluted materials were subsequently analyzed by Western blotting.

### Viral infectivity assay

Viral infection was determined by a multinuclear activation of a galactosidase indicator (MAGI) assay as described previously [12]. Briefly, MAGI-CCR-5 cells were seeded in 24-well plates 1 day before infection. The MAGI-CCR-5 cells were infected at 20–30% confluency. Virus input was normalized by the level of p24. Equal p24 units of virus samples were mixed with 20 μg/ml DEAE-dextran and incubated with MAGI-CCR5 cells for 2 h. The initial infection period was terminated by addition of fresh DMEM. After incubation for 48 h at 37°C in a 5% CO2 incubator, supernatants were removed, and the cells were fixed with 500 μl of fixing solution (1% formaldehyde and 0.2% glutaraldehyde in PBS) for 5 min and stained with 5-bromo-4-chloro-3-indolyl-β-D-galactopyranoside (X-Gal). As β-galactosidase activity is under the control of the HIV-1 long terminal repeat (LTR) promoter in this system, positive blue dots representing β-galactosidase activity were counted to determine viral infectivity.

### Sequence analysis and modeling

The homology model of BIV Vif was built by Discovery Studio 2.1 software package using the crystal structure of tatD DNase of *E. Coli.* (PDB ID: 1XWY) as the template. Modeling was performed at the medium optimization level with refined loop parameters, and no additional restraints were used. Ten models were built, and the model with the best score was chosen.

## Abbreviations

BIV: Bovine immunodeficiency virus
MVV: Maedi-visna virus
Vif: Virus infectivity factor
bt: Bos taurus
oa: Ovis aries
Cul2: Cullin2
Cul5: Cullin5
EloB/EB: ElonginB
EloC/EC: ElonginC
CBF-β/CBF: Beta core binding factor beta
EloCΔ2/ECΔ2: ElonginC mutant with replacement of critical hydrophobic amino acids A100 and L103 with hydrophilic serine
Cul2/5ΔNedd8: Nedd8-negative Cullin2/5 mutant
